# Identification and Pathogenicity of a New Entomopathogenic Fungus, *Mucor hiemalis* (Mucorales: Mucorales), on the Root Maggot, *Bradysia odoriphaga* (Diptera: Sciaridae)

**DOI:** 10.1093/jisesa/ieac010

**Published:** 2022-03-18

**Authors:** Guodong Zhu, Wenjuan Ding, Ming Xue, Yongfei Zhao, Mingzhu Li, Zizheng Li

**Affiliations:** 1 College of Agronomy, Liaocheng University, Shandong Province, 252000, China; 2 College of Plant Protection, Shandong Agricultural University, Tai’an City, Shandong Province, 271018, China; 3 Liaocheng Academy of Agricultural Sciences, Liaocheng, 252000, China

**Keywords:** entomopathogenic fungi, *Bradysia odoriphaga*, biocontrol agent, pathogenicity bioassay, control efficiency

## Abstract

*Bradysia odoriphaga* Yang and Zhang (Diptera: Sciaridae), the Chinese chive root maggot, is a destructive pest of *Allium* vegetables and flowers that causes severe losses in northern China. Novel biological control technologies are needed for controlling this pest. We identified a new entomopathogenic fungus isolated from infected *B. odoriphaga* larvae and evaluated the susceptibility of the biological stages of *B. odoriphaga* and the effects of temperature on fungus growth and pathogenicity. Based on morphological characteristics and molecular phylogeny, the fungus was identified as *Mucor hiemalis* BO-1 (Mucorales: Mucorales). This fungus had the strongest virulence to *B. odoriphaga* larvae followed by eggs and pupae, while *B. odoriphaga* adults were not susceptible. A temperature range of 18–28°C was optimum for the growth and sporulation of *M. hiemalis* BO-1 and virulence to *B. odoriphaga* larvae. At 3 and 5 d after inoculation with 10^5^ spores/ml at 23°C, the survival rates were 24.8% and 4.8% (2nd instar larvae), respectively, and 49.6% and 12.8% (4th instar larvae), respectively. The potted plant trials confirmed that *M. hiemalis* BO-1 exerted excellent control efficiency against *B. odoriphaga* larvae, and the control exceeded 80% within 5 d when the spore concentration applied exceeded 10^7^ spores/ml. In conclusion, these findings supported the hypotheses that this fungus could serve as an effective control agent against *B. odoriphaga* larvae and is worth being further tested to determine its full potential as a biocontrol agent.

## Graphical Abstract

**Figure:**
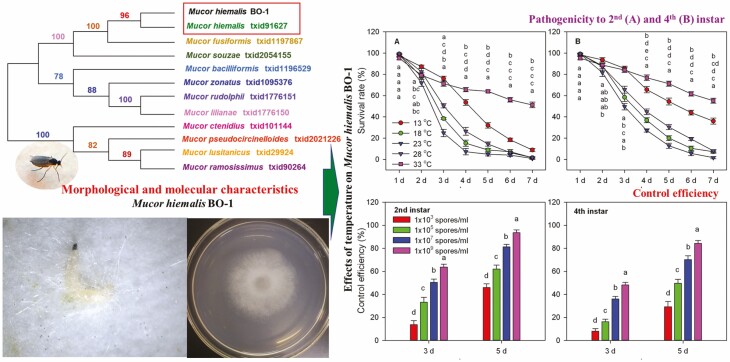

Biological control is an important component of Integrated Pest Management (IPM), owing to its reduced impact on the environment and its substantial efficacy (Shah et al. 2003, Hajek et al. 2007). The biological control agents of pests include predators, parasitic insects, entomopathogenic fungi, entomopathogenic bacteria, insect viruses, and entomopathogenic nematodes (Lacey et al. 2001). Entomopathogenic fungi (EPF) comprise a diverse group that includes potential biocontrol agents of many destructive pest species (Bale et al. 2008, Gabarty et al. 2012). Several entomopathogenic fungi have been used for pest control, including *Beauveria bassiana* (Balsamo), *Metarhizium anisopliae* (Metschnikoff), *Nomuraea rileyi* (Farlow), *Verticillium lecanii* (Zimmerman), and *Paecilomyces fumosoroseus* (Wize) (Coombes et al. 2016). However, despite the great variety of entomopathogenic fungi, only a few species have been successfully developed as biopesticides. *Beauveria bassiana* and *Metarhizium anisopliae* are potential candidates for the biological control of many agricultural pests, such as Lepidoptera, Coleoptera, and Diptera (Kassa et al. 2004, Khoury et al. 2019).

*Bradysia odoriphaga* Yang and Zhang (Diptera: Sciaridae), the Chinese chive root maggot, is a serious pest of *Allium* vegetables and oramental flowers in northern China (Yang and Zhang 1985, Xue et al. 2005). The larvae tend to aggregate in fields and directly damage plants by feeding on root and corm tissues, resulting in wilt or rot (Zhang et al. 2016). Since the damage caused by *B. odoriphaga* larvae is cryptic and primarily occurs on the underground portions of plants, it is difficult to effectively control (Zhu et al. 2017, Shi et al. 2018). To date, the most common management practice for *B. odoriphaga* larvae is applications of organophosphates, carbamates, and neonicotinoid insecticides (Ma et al. 2013). However, after many years of treatments, larvae have developed resistance to pesticides, and this has resulted in unsatisfactory control (Bai et al. 2016a). In addition, conventional chemical pesticide use has been increasingly restricted due to environmental pollution and human health concerns (Shan et al. 2020). Therefore, exploring biological control resources is important for reducing pesticide applications, decreasing environmental pollution, and improving pest population management.

Previous studies reported that compared to other agricultural pests, there are few applicable biological control agents of *B. odoriphaga*. These include *Beauveria bassiana* (entomopathogenic fungus), *Bacillus thuringiensis* (entomopathogenic bacteria), *Stratiolaelaps scimitus* (predatory mite), and *Steinernema feltiae* (entomopathogenic nematodes) (Chen et al. 2016, Zhou et al. 2014). Song et al. (2016) isolated a *Beauveria thuringiensis* strain JQ23 from soil, possessing high insecticidal activity against *B. odoriphaga*. This strain had a 72 hr LC_50_ of 8.38 × 10^6^ spore/ml against 2nd instar larvae. Only *Beauveria bassiana*, *Bacillus thuringiensis*, and entomopathogenic nematodes have been applied to control *B. odoriphaga* in the field (Chen et al. 2016). Zhou et al. (2014) reported that when the dosage of *Beauveria bassiana* granules (15 billion spore/g) was 4.5 kg/ha, the field control efficiency was 81.15% (7 d after application) and 85.19% (21 d after application). Although many entomopathogenic fungi possess good pathogenicity in the laboratory, their field efficacy depends on favorable environmental conditions, such as temperature, humidity, and characteristics of the microorganism (Ekesi et al. 1999, Fargues et al. 2003). For example, most entomopathogenic fungi exert good pathogenicity at optimum temperatures (Mwamburi et al. 2015), and the optimum temperatures of *Beauveria bassiana* that provided the best control efficiency ranged from 25 to 30°C (Fargues et al. 1997, Shimazu 2004). *Bradysia odoriphaga* is an underground insect in its immature stages and adults leave the soil. However, during the emergence peak of *B. odoriphaga*, the mean soil temperature in fields is typically below 20°C, which is not optimal for many biological insecticides (Shi et al. 2020). To sum up, finding an entomopathogenic fungus possessing high pathogenicity and broad environmental adaptability for use against *B. odoriphaga* appears to be key to improving biocontrol efficiency.

The genus *Mucor* includes many species of filamentous fungi widely distributed in soil that are usually associated with abundant humus. Most species are saprotrophic and widely applied in fermentation processes. Individual *Mucor* species are well known as pathogens of animals and humans, although there are a few *Mucor* species reported as insect pathogens. Reiss et al. (1993) reported that some *Mucor hiemalis* strains were pathogenic to *Artemia salina*, an important arthropod. Konstantopoulou and Mazomenos (2005) isolated six species of entomopathogenic fungi from diseased *Bactrocera oleae* and *Sesamia nonagrioides*. Bioassays revealed that two strains of *M. hiemalis* were pathogenic to *Ceratitis capitata* larvae causing more than 80% mortality. This is the only report concerning the pathogenicity of *M. hiemalis* to Diptera.

In 2019, we isolated one strain of entomopathogenic fungi from infested *B. odoriphaga* larvae collected from a Chinese chive field. Morphological observation indicated that this strain was a *Mucor* species. In the process of culture, this strain was strongly pathogenic and infectious, causing laboratory population extinction within a few days once spore transmission spread occurred. We suspected that this entomopathogenic fungus had the potential for development as a biocontrol agent.

In this study, we identified the isolate from infected *B. odoriphaga* larvae using morphological and molecular analyses. The susceptibility of the life stages of *B. odoriphaga* to this entomopathogenic strain and the effects of temperatures on fungus growth and pathogenicity were evaluated. Additionally, the control efficiency was also conducted by a potted plant trial. Our results provide a new biological control agent for application in *B. odoriphaga* control and increase available options for exploring the green control techniques for crop root maggots.

## Materials and Methods

### Insect Materials

Samples of *B. odoriphaga* larvae were originally collected from a Chinese chive field in Tai’an, Shangdong, in April 2015. The samples were maintained at the College of Agronomy, Liaocheng University, and reared on Chinese chives for more than 10 generations using the method described by Xue et al. (2005). Eggs, larvae, and pupae were reared in culture dishes (Φ = 9 cm), and pairs of newly emerged adults were placed in plastic oviposition containers (3 cm diameter × 1.5 cm high). Colonies were maintained in growth cabinets at 23 ± 1°C with 75 ± 5% relative humidity.

### Entomopathogenic Fungi Isolated From Infested Larvae

Infested *B. odoriphaga* larvae were collected in winter from Chinese chives field in Liaocheng, Shangdong (N 36.784, E 115.447, 10 November 2019). According to the method described by Barra et al. (2013), all symptomatic *B. odoriphaga* larvae with signs of fungal infection were surface sterilized with a 1% sodium hydrochloride solution dip for 1 min. Subsequently larvae were washed three times with distilled water and placed on Potato Dextrose Agar (PDA) plates (Φ = 9 cm). Plates were incubated at 23°C for 3 d to obtain growth of mycelia occurring internally, and the hypha at the edge of the colony was picked and transferred to new PDA plates to obtain a pure strain.

### Identification of Entomopathogenic Fungi Isolates

#### Morphological Description

The pure entomopathogenic fungus strain was incubated at 23°C, and the microscopic features (morphology of hyphae, fruiting bodies, and spores) and macroscopic features (colony morphology and color) were examined by microscopic observation methods described by Nyongesa et al. (2015) andGerald (1995).

#### Molecular Phylogenetic Analysis

DNA from a fungal isolate was extracted using an EasyPure Fungi Genomic DNA Kit (ComWin Biotech, Beijing, China). Sequences containing the region encoding ITS was PCR amplified with primers described by Hinrikson et al. (2005) (ITS4: 5′-TCCTCCGCTTATTGATATGC-3′, ITS5:5′-GGAAGTAAAAGTCGTAACAAGG-3′). The fungal DNA was sequenced by BioSune Limited Company (Shanghai, China). Resulting ITS sequences were aligned using ClustalW in Mega v. 6.0, and their homologies were determined by BLAST searches within the NCBI (National Center for Biotechnology Information) database. A phylogenetic tree of the isolate was constructed using model strains of genus *Mucor hiemalis* (Taxonomy ID: 91627, Accession: NR_152948.1), *Mucor fusiformis* (Taxonomy ID: 1197867, Accession: NR_111660.1), *Mucor souzae* (Taxonomy ID: 2054155, Accession: NR_165210.1), *Mucor bacilliformis* (Taxonomy ID: 1196529, Accession: NR_145285), *Mucor zonatus* (Taxonomy ID: 1095376, Accession: NR_103638), *Mucor rudolphii* (Taxonomy ID: 1776151, Accession: NR_152977), *Mucor lilianae* (Taxonomy ID: 1776150, Accession: NR_152978), *Mucor ctenidius* (Taxonomy ID: 101144, Accession: NR_168144), *Mucor pseudocircinelloides* (Taxonomy ID: 2021226, Accession: NR_169896), *Mucor lusitanicus* (Taxonomy ID: 29924, Accession: NR_126127), and *Mucor ramosissimus* (Taxonomy ID: 90264, Accession: NR_103627). This phylogenetic tree was constructed with MEGA v7.0, by the neighbor-joining method.

### Effects of Environmental Temperatures on Fungus Growth

Pure fungal isolate agar disks (Φ = 8 mm) containing mycelium were made by a fungal puncher from the edge of the pure colony. Every agar disk was placed upside down at the center of a PDA plate (Φ = 9 cm). The plates were incubated in growth chambers at different temperatures (13, 18, 23, 28, and 33°C) according to a study on the effects of temperature gradients on *B. odoriphaga* (Li et al. 2015). At 23°C temperature was used as the control. Every temperature treatment contained five plates (replicates). The colony diameter was recorded daily using the cross-bonded method. Colony growth was recorded as mean perpendicular radius minus the diameter of the inoculum plug. After 10 d, the spore suspension of every plate was prepared using 50 ml 0.1% Tween 80 distilled water according to the description by Mwamburi et al. (2015), and the spore suspension concentration was calculated with blood counting chamber analysis.

### Pathogenicity Bioassays

#### Pathogenicity to Biological Stages of *B. odoriphaga*

Serial spore suspension dilutions (1 × 10^3^, 1 × 10^5^, and 1 × 10^7^ spores/ml) were made with 0.1% Tween 80 distilled water. Eggs, 2nd and 4th instar larvae, pupae, and adults were used as the test subjects. Bioassays on 2nd and 4th instar larvae (within 1 d after molt) were conducted using standard contact and stomach bioassay methods with slight modifications (Zhang et al. 2016). One piece of filter paper (Φ = 9 cm) was moistened by dropping 1 ml of spore suspension and placed in a culture dish, and fresh diet (fresh Chinese chives) was placed on the filter paper. Larvae were placed around the fresh diets. Eggs, pupae, and adults were reared in culture dishes (Φ = 9 cm) covered with filter paper moisturized by 1 ml spore suspension. This bioassay was conducted at 23°C, and pure water treatment was used as the control. Each treatment (concentration) contained five replicates and each replicate contained 20 individuals. *B. odoriphaga* mortality was recorded daily.

#### Pathogenicity Under Different Temperatures

According to the results of pathogenicity to biological stages, spore suspension (1 × 10^5^ spores/ml) possessing moderate pathogenicity to *B. odoriphaga* larvae was chosen as the test dosage. The spore suspensions (1 × 10^5^ spores/ml) were made with 0.1% Tween 80 distilled water. Bioassays on 2nd and 4th instar larvae were conducted using a previously described method. The culture dishes after adding spore suspension and test larvae were transferred to different temperature conditions (13, 18, 23, 28, and 33°C). Each treatment contained five replicates and each replicate contained 20 individuals. Larval survival was recorded daily for 7 d.

### Trials with Potted Plants

Serial spore suspension dilutions (1 × 10^3^, 1 × 10^5^, 1 × 10^7,^ and 1 × 10^9^ spores/ml) were made with 0.1% Tween 80 distilled water. The 2nd and 4th instar larvae of *B. odoriphaga* were used as the test subjects. Potted Chinese chives (Xuejiu Variety) planted for two years were prepared. Pots with no larval damage were chosen as the experimental material. Before the trial, the aboveground part of Chinese chives was cut off and 2nd or 4th instar larvae were transferred into the rhizosphere soil. On the day of the experiment, 25 ml of spore suspension was added to the soil of every pot. Pure water (without spores) treatment was used as the control. Each treatment contained five replicates and each replicate contained 40 individuals. During the experiment, adequate water was added daily to maintain soil moisture. All the pots were maintained in growth chambers at 23 ± 1°C with 75 ± 5% relative humidity, and a 12:12 hr light: dark cycle. On the 3rd and 5th day after treatment, the larval survival of *B. odoriphaga* was recorded. The control efficiency was calculated according to Equations (1) and (2):


$$\begin{array}{l} Survival\;rate=\;\;\;\frac{The\;\;\;number\;\;\;of\;\;\;survival\;\;\;insects}{The\;\;\;total\;\;\;number\;\;\;of\;\;\;tested\;\;\;insects}\times 100\% \end{array}$$ (1)

$$\begin{array}{l} Control\;ef\;f\;iciency\;\;\;=\frac{Survival\;\;\;rate\;\;\;of\;\;\;control\;\;\;group-Survival\;\;\;rate\;\;\;of\;\;\;treatment\;\;\;group}{100-Survival\;\;\;rate\;\;\;of\;\;\;control\;\;\;group}\times 100\% \end{array}$$ (2)

### Statistical Analysis

Statistical analysis was performed using SPSS statistics software (Version 19.0, SPSS, Chicago, IL). Values in the heat hardening treatments were compared by one-way ANOVA followed by Tukey’s HSD multiple comparison test (*P* < 0.05).

## Results

### Morphological and Molecular Characteristics

#### Macroscopic Morphological Analysis

**Fig. 1.**
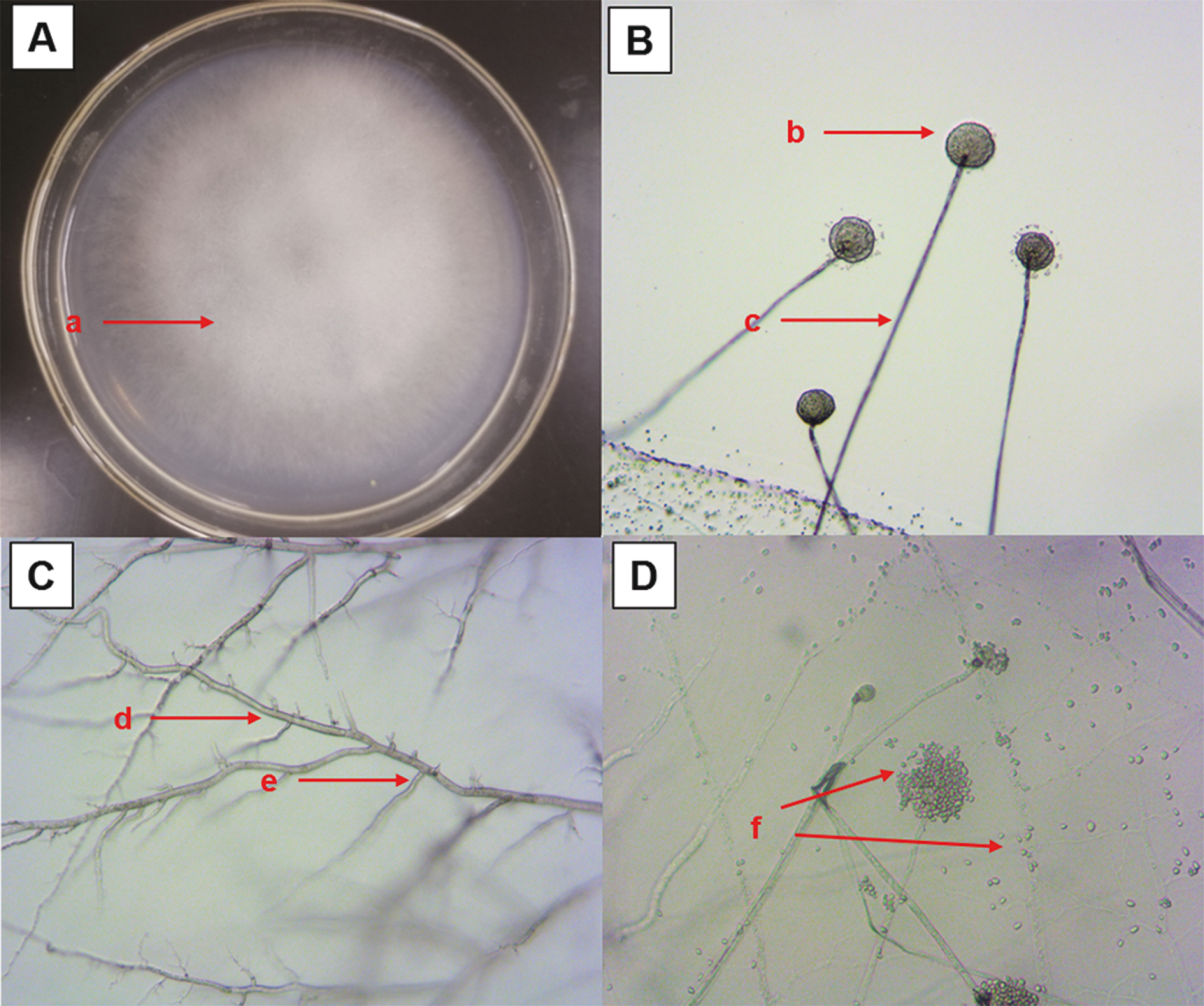
Macroscopic morphological characteristics of *Mucor hiemalis* BO-1. A (a) white and slightly transparent aerial hyphae on potato dextrose agar (PDA) medium; B (b) small black sporangium; (c) vertical and uniramous sporangiophore; C (d) smooth, aseptate and polynuclear hyphae; (e) branched hyphae; D (f) broken sporangium releasing sporangiospores.

This entomopathogenic fungi colonies cultured on PDA were white and slightly transparent, covered with thick long pile like cotton on the side and a rough surface (aerial hyphae) (Fig. 1A). According to the description by Gerald (1995), small black dots (sporangium) were borne on the tip of the mycelium (Fig. 1A). Microscopic characterization of *Mucor hiemalis* BO-1 using an optical microscope showed that the hyphae were smooth, aseptate, and polynuclear (Fig. 1C). The sporangiospores were globose to subglobose with smooth outer walls and wrapped in the sporangia (Fig. 1D), which were subglobose and at the top of individual aseptate hyphae (Fig. 1B).

#### Molecular Phylogenetic Analysis of ITS Region

**Fig. 2.**
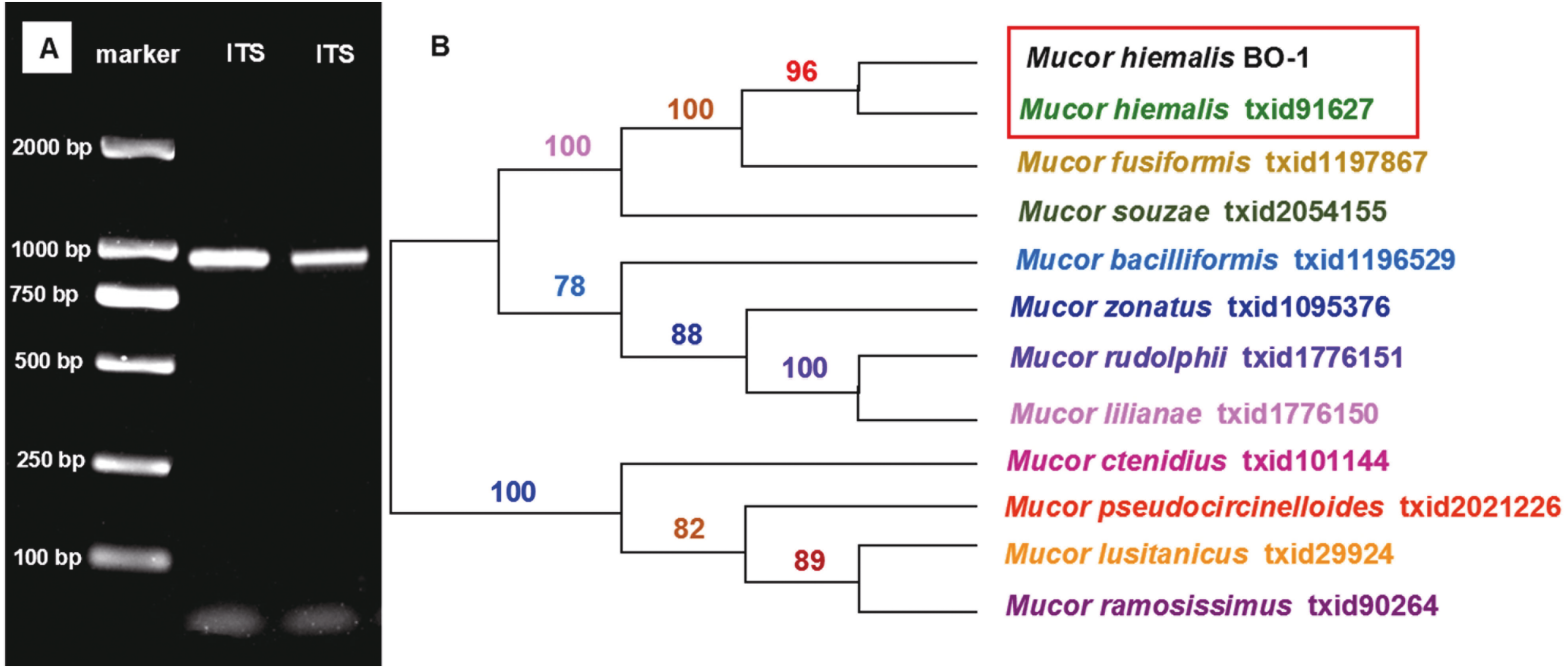
Neighbor-joining tree based on the analysis of partial ITS sequences of *Mucor hiemalis* BO-1 and other *Mucor* species. A, The electrophoretic band of *Mucor hiemalis* BO-1 partial ITS sequence. B, neighbor-joining tree.

The ITS rDNA region of *Mucor hiemalis* BO-1 was amplified and sequenced. The length of the ITS sequence was 936 bp and it was deposited in the GenBank database (accession number MN686205). The sequence similarity search using ITS sequences of *Mucor hiemalis* BO-1 in BLASTN program revealed that this strain and *Mucor hiemalis* (CBS201.65) were present in the same cluster with 96% sequence similarity, which were well supported by a bootstrap value of 100% (Fig. 2).

### Pathogenicity of *Mucor hiemalis* BO-1 Against *B. odoriphaga* Larvae

**Fig. 3.**
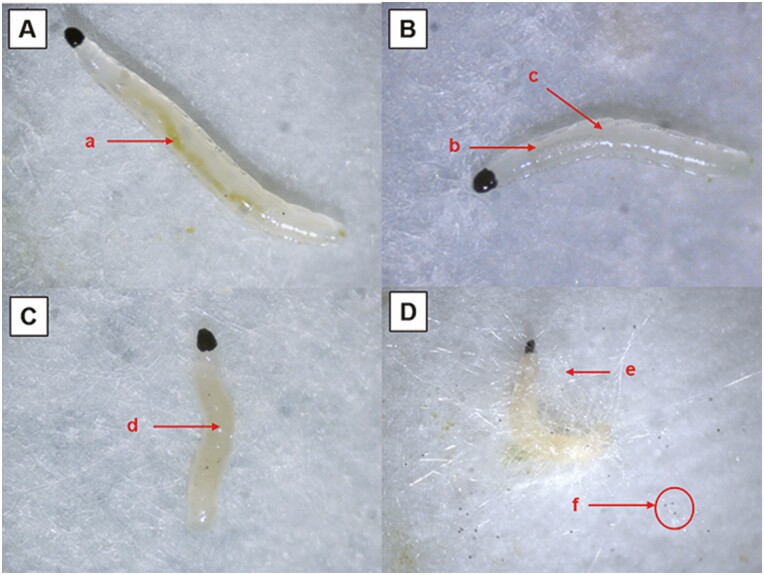
Pathogenesis of *B. odoriphaga* larvae infected by *Mucor hiemalis* BO-1. A 24 hr after inoculation; (a) green food particles were present in larval guts; B 48 hr after inoculation; (b) few food particles were present in larval guts; (c) the body gradually became transparent and bright; C 72 hr after inoculation; (d) yellow, turbid and supple larval body; D 120 hr after inoculation; (e) numerous white aerial hyphae grew from the dead larval body; (f) black sporangia at the top of the mycelium.

A total of 100 *B. odoriphaga* larvae treated with *Mucor hiemalis* BO-1 (1.0 × 10^6^ spores/ml) were raised in moist petri dishes and observed with a stereoscopic microscope. In the early stages of infection (24 hr after inoculation), the larval movement became slower. Few food particles were present in larval guts (Fig. 3A). In the mid-course of pathogenesis (48 hr after inoculation), larval behavior was more sluggish, and the larvae barely moved even after being touched by brush. The larval body became transparent gradually. At 48 hr, the larval feeding capacity was maintained, but the food consumption declined dramatically and only a small amount of diet material was seen in the digestive tract (Fig. 3B). At the end of pathogenesis (72 hr after inoculation), the larvae were moribund (only the head and mouthparts moved slightly) and the body was yellow, turbid, and supple (Fig. 3C). In the growth period of the hyphae (120 hr after inoculation), numerous white aerial hyphae grew from the dead larval body, and black sporangia were present at the top of the mycelium (Fig. 3D).

### Pathogenicity of *M. hiemalis* BO-1 to Biological Stages of *B. odoriphaga*

**Fig. 4.**
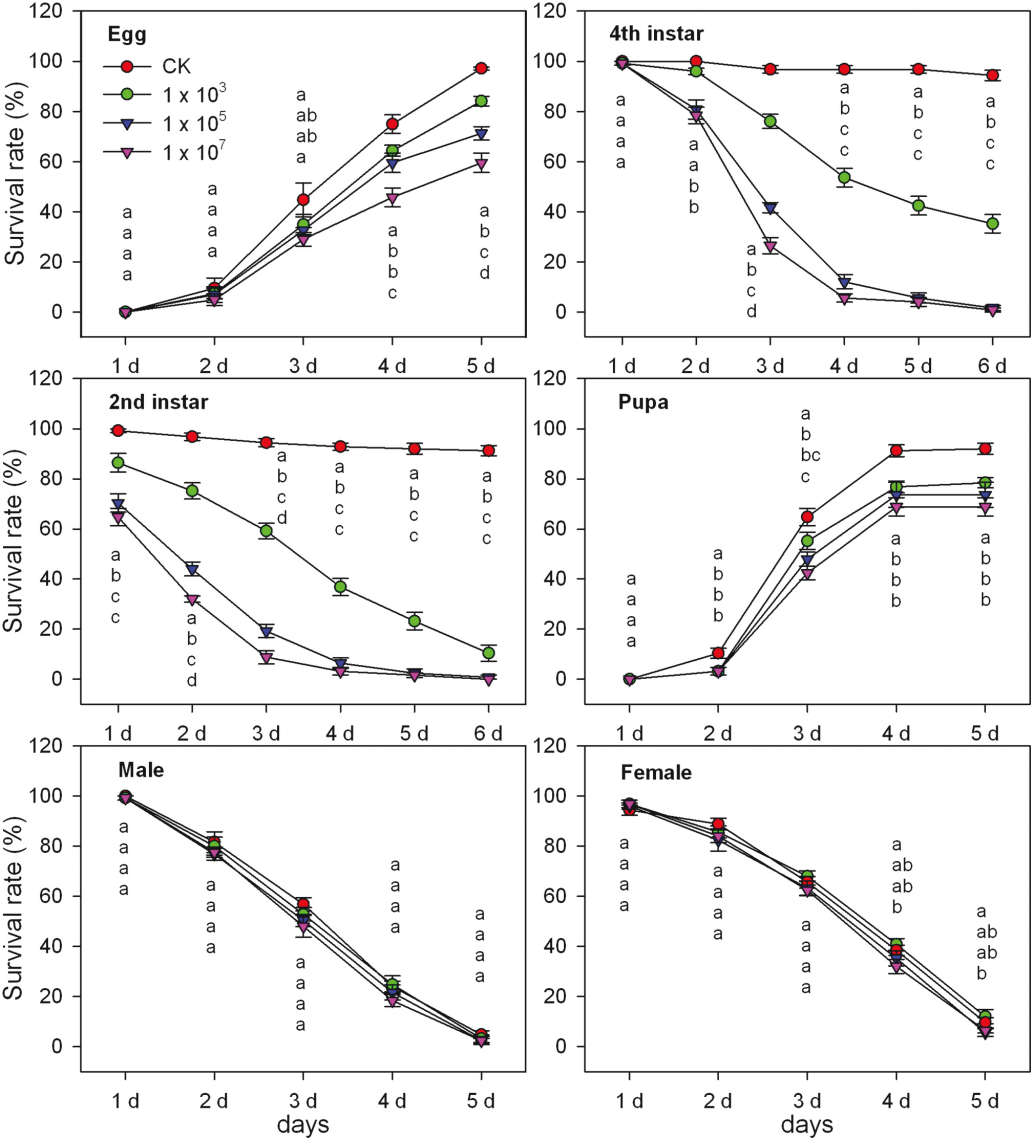
**Pathogenic effects of *Mucor hiemalis* BO-1 on different stages of *Bradysia odoriphaga*.** Data in the figure are the mean ± SE. Different letters over the same column indicate significant differences between different spore concentration treatments at the *P* < 0.05 level as indicated by one-way ANOVA.

We observed the infection of *M. hiemalis* BO-1 on different stages of *B. odoriphaga*, and the bioassay results indicated that *M. hiemalis* BO-1 was more pathogenic effect to *B. odoriphaga* larvae than to eggs, pupae, and adults (Fig. 4). For 2nd instar larvae, the survival rates after inoculation decreased to 75.2% (10^3^ spores/ml), 44% (10^5^ spores/ml), and 32% (10^7^ spores/ml), while that of the control was 96.8%. With the extension of time, the survival rates are declined rapidly. At 4 d after inoculation, the survival rates were 36.8% (10^3^ spores/ml), 6.4% (10^5^ spores/ml), and 3.2% (10^7^ spores/ml). The 4th instar larvae were less susceptible to *M. hiemalis* BO-1 than the 2nd instar larvae. At 4 d and 5 d after treatment, the survival rates were 53.6% and 42.4% (10^3^ spores/ml), respectively, 12% and 5.6% (10^5^ spores/ml), respectively, and 5.6% and 4% (10^7^ spores/ml), respectively. These rates were higher than those of the 2nd instar larvae.

*M. hiemalis* BO-1 showed only slight pathogenicity to *B. odoriphaga* eggs and pupae. At 5 d after treatment, the egg hatchability was 84.12% (10^3^ spores/ml), 71.29% (10^5^ spores/ml), and 59.51% (10^7^ spores/ml), and most newly hatched larvae died within 1 d. The adult emergence rates were 78.4% (10^3^ spores/ml), 73.6% (10^5^ spores/ml), and 68.8% (10^7^ spores/ml). *B. odoriphaga* adults were scarcely affected based on no significant differences in longevity among the different treatments.

### Effects of Temperature on the Growth and Pathogenicity of *Mucor hiemalis* BO-1

#### Fungus Growth

**Fig. 5.**
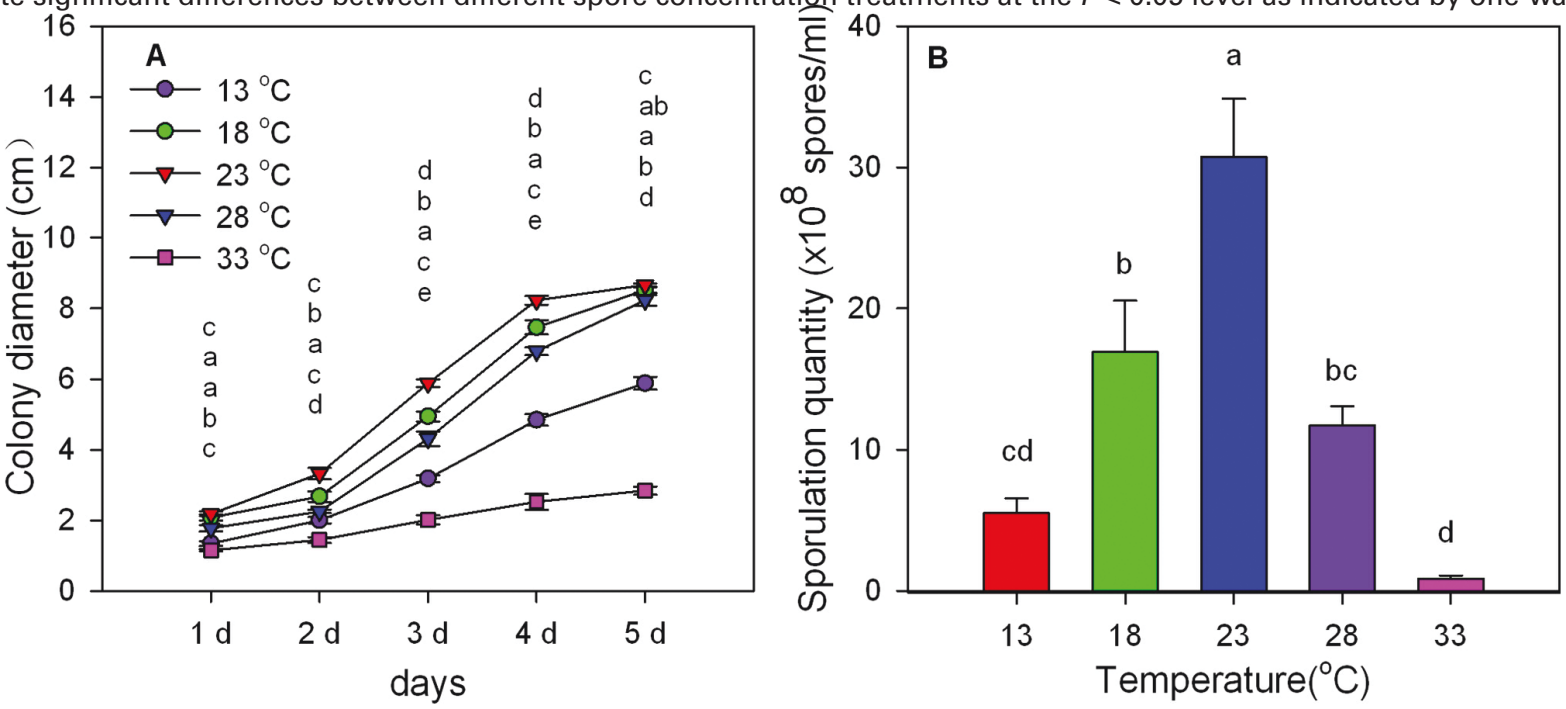
Effects of temperature on mycelial growth (A) and conidiation (B) of *Mucor hiemalis* BO-1. Data in the figure is the mean ± SE. Different letters over the same column from the top indicate significant differences between different temperature treatments at the *P* < 0.05 level as indicated by one-way ANOVA.

The culture results showed that the growth and sporulation quantity of *M. hiemalis* BO-1 were affected by temperature (Fig. 5). Temperatures ranging from 18 to 28°C were beneficial to mycelial growth, while the mycelium grew poorly at 33°C (Fig. 5A). After 5 d culture, the colony diameter was 5.83 cm (13°C), 8.52 cm (18°C), 8.66 cm (23°C), 8.23 cm (28°C), and 2.84 cm (33°C) (*F*_4,20_ = 398.397, *P* < 0.001). After 10 d culture, the sporulation quantities were 5.5 × 10^8^ spores/ml (13°C), 1.69 × 10^9^ spores/ml (18°C), 3.07 × 10^9^ spores/ml (23°C), 1.17 × 10^9^ spores/ml (28°C), and 0.86 × 10^8^ spores/ml (33°C), and there were significant differences between different treatments (*F*_4,20_ = 19.969, *P* < 0.001) (Fig. 5B). We found that 23°C was the optimum temperature for growth and sporulation of *M. hiemalis.*

#### Pathogenicity

**Fig. 6.**
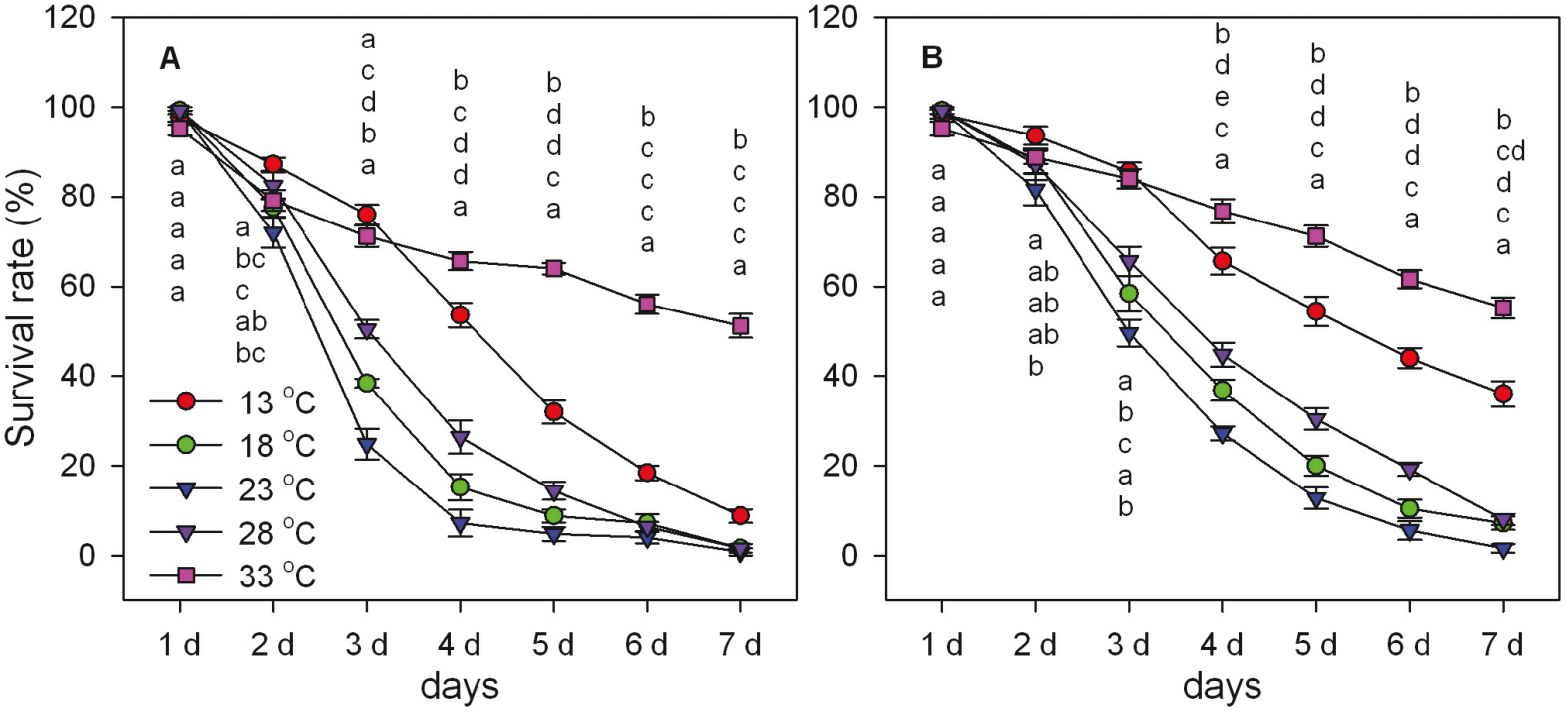
Effects of environmental temperatures on the pathogenicity of *Mucor hiemalis* BO-1 to *Bradysia odoriphaga* 2nd (A) and 4th (B) instar larvae. Data in the table are the mean ± SE. Different letters over the same column from the top indicate significant differences between different temperature treatments at the *P* < 0.05 level as indicated by one-way ANOVA.

The bioassay results indicated that the temperature affected the pathogenicity of *M. hiemalis* BO-1 to *B. odoriphaga* larvae and temperatures ranging from 18 to 28°C were the most effective for *M. hiemalis* BO-1 (Fig. 6). For 2nd instar larvae, at 3 d after inoculation with 10^5^ spores/ml, the survival rates were 38.4% (18°C), 24.8% (23°C), and 50.4% (28°C). These rates were significantly lower than survival at 13°C (76%) and 33°C (71.2%). For 4th instar larvae, at 3 d after inoculation with 10^5^ spores/ml, the survival rates were 58.4% (18°C), 49.6% (23°C), and 65.6% (28°C). These were significantly lower than survival rates at 13°C (85.6%) and 33°C (84%). At 5 d after inoculation, a similar data trend was observed. The relatively high 33°C temperature might suppress the pathogenicity of *M. hiemalis* BO-1. The survival rates were still 55.2% (4th instar) and 51.2% (2nd instar) until 7 d, and the mortality might have been affected by high temperature. The optimum temperature for the pathogenicity of *M. hiemalis* BO-1 was 23°C followed by 18°C and 28°C.

### Results of Potted Plant Trials

**Fig. 7.**
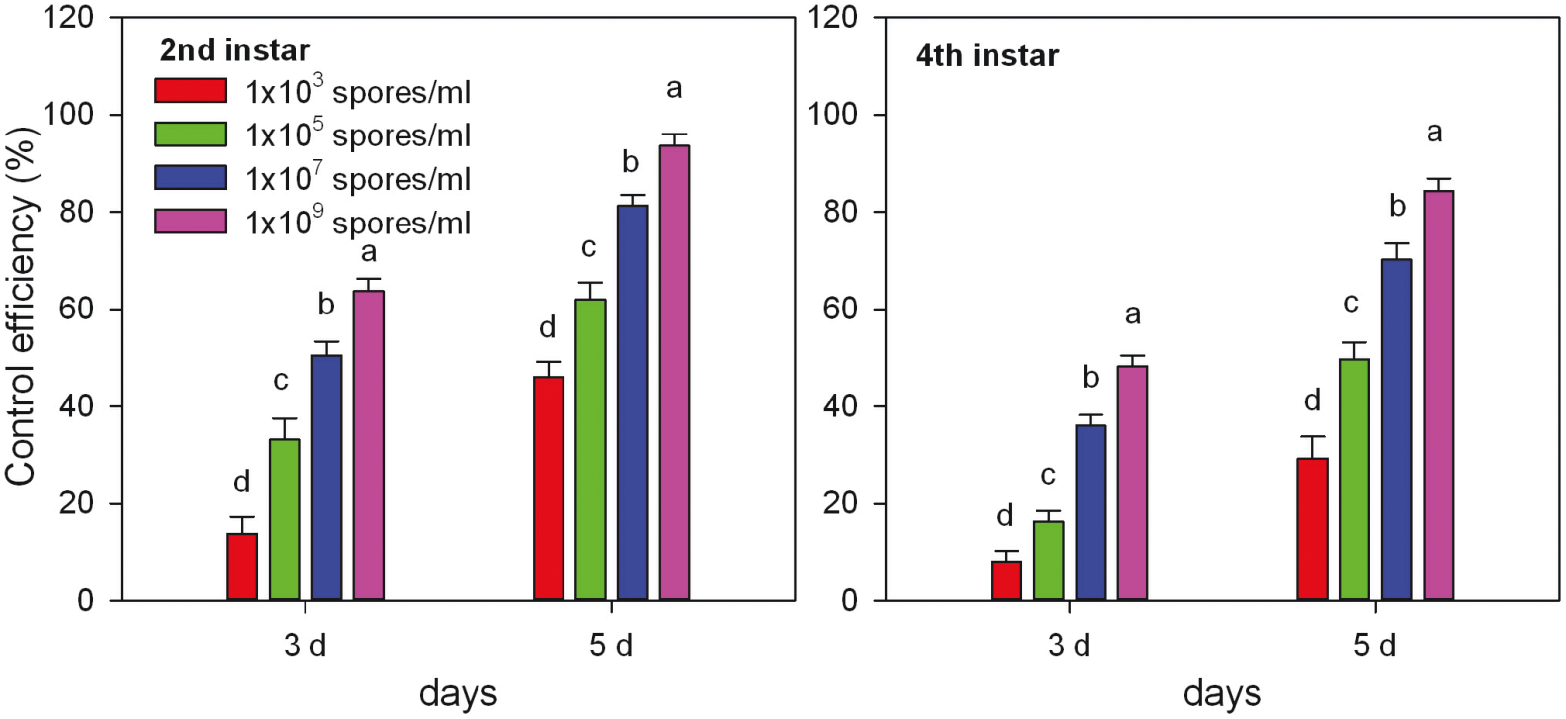
Control efficacy of *Mucor hiemalis* BO-1 against *Bradysia odoriphaga* 2nd and 4th instar larvae. Data in the table are the mean ± SE. Different letters over the same column from the top indicate significant differences between different treatments at the *P* < 0.05 level as indicated by one-way ANOVA.

*M. hiemalis* BO-1 provided superior control of *B. odoriphaga* 2nd and 4th instar larvae when the spore suspension concentration exceeded 10^7^ spores/ml. At 3 d after the 2nd instar larvae treatment, the control efficiencies were 33.16% (1 × 10^5^ spores/ml), 50.51% (1 × 10^7^ spores/ml), and 63.77% (1 × 10^9^ spores/ml) (Fig. 7). For 4th instar larvae, the control efficiencies were 16.25% (1 × 10^5^ spores/ml), 36.04% (1 × 10^7^ spores/ml), and 48.22% (1 × 10^9^ spores/ml), respectively. At 5 d after the 2nd instar larvae treatment, the control efficiencies were 45.99% (1 × 10^3^ spores/ml), 62.03% (1 × 10^5^ spores/ml), 81.28% (1 × 10^7^ spores/ml), and 93.58% (1 × 10^9^ spores/ml), respectively (Fig. 7). For 4th instar larvae, the control efficiencies were 29.32% (1 × 10^3^ spores/ml), 49.74% (1 × 10^5^ spores/ml), 70.16% (1 × 10^7^ spores/ml), and 84.29% (1 × 10^9^ spores/ml), respectively.

## Discussion

In this study, we isolated a new entomopathogenic fungus strain from infested *B. odoriphaga* larvae. Based on morphological and molecular characters, this strain was identified as *Mucor hiemalis*. The unique morphological features of this strain include white colonies covered with fluffy mycelia and globose sporangia on the tips of the mycelium. These features revealed that this isolate was *Mucor hiemalis* (Zhang et al. 2018). Molecular identification (ITS sequence analysis) with supplementary elements was performed to confirm the identification (Ardeshir et al. 2016). Reiss et al. (1993) reported the pathogenicity of 15 species of Mucorales (including *M. hiemalis*) to brine shrimp (*Artemia salina*) larvae, andBibbs et al. (2013) reported that a new stain of *Mucor fragilis* Bainier had strong pathogenicity to brown widow spiders (*Latrodectus geometricus* Koch). Therefore, this entomopathogenic fungi strain was named *Mucor hiemalis* BO-1.

Biological assays revealed that *Mucor hiemalis* BO-1 had a substantial pathogenic effect on *B. odoriphaga* larvae. After infection, *B. odoriphaga* larval movement slowed, and the insect body became transparent. During infection, food residue in the alimentary canal decreased. Therefore, it appears that *Mucor hiemalis* BO-1 infection disrupted the physiological and digestive functions of *B. odoriphaga* because of nutritional plunder, degrading enzymes, and toxic proteins. Previous studies have reported that *Beauveria bassiana* and *Metarhizium anisopliae* produce insecticidal mycotoxins against pests (Sowjanya et al. 2008, Khoury et al. 2019, Yin et al. 2021). Wei et al. (2017) reported that *Beauveria bassiana* infection resulted in dysbiosis of mosquito gut microbiota and decreasing bacterial diversity, which promoted mortality of the mosquito larvae. Most entomopathogenic fungi gain access to the body cavity through the external cuticle, where they consume nutrients, produce toxins, destroy host cells and eventually kill the hosts (Wang and Wang 2017). A few entomopathogenic fungi invade the body through the digestive tract by direct feeding of insects, where they disrupt larval feeding capacity primarily during the initial stage of pathogenesis (Kubicek and Druzhinina 2007). However, determination of the main infection mode for *Mucor hiemalis* BO-1 against *B. odoriphaga* larvae will require additional studies.

Entomopathogenic fungi have been considered as biopesticides because they are an environmentally friendly alternative to chemical insecticides (Shah and Pell 2003, Barra et al. 2013). Clarifying the susceptibility of biological stages of pests to entomopathogenic fungi is crucial for field application and this information be used to effectively target the most damaging insect stages (Shah and Pell 2003,Angel et al. 2005). The susceptibility bioassay results showed that *B. odoriphaga* larvae are more susceptible to *M. hiemalis* BO-1 than other life stages, and early-instar larvae are more susceptible than older larvae. In addition, *M. hiemalis* BO-1 also showed mild pathogenicity to eggs and pupae. At 120 hr after treatment with 10^7^ spores/ml, the survival rate was 59.51% (eggs) and 68.8% (pupae). Although some larvae from treated eggs successfully eclosed, they died within 24 hr. This may have resulted from the strong pathogenicity of *M. hiemalis* BO-1 to early-instar larvae. However, *B. odoriphaga* adults were not susceptible to *M. hiemalis BO-1*. In contrast, Daniel and Wyss (2009) observed higher mortality of *Rhagoletis cerasi* adults and larvae in contact bioassay for all the fungi (*Beauveria bassiana*, *Metarhizium anisopliae*, and *Isaria fumosorosea*) tested as compared to the other life stages, and the adult life span of *R. indifferens* treated with *M. anisopliae* was shortened and fertility was reduced. Yee and Lacey (2005) also reported that *Rhagoletis indifferens* adults were more susceptible to *Metarhizium anisopliae* than larvae and eggs. These dissimilar results could be caused by differences among the entomopathogenic fungi species, which possess different infection modes, or different types of insect species. In this study, *M. hiemalis* BO-1 exhibited strong pathogenicity to *B. odoriphaga* larvae, which is the stage causing the greatest plant damage and economic losses (Zhang et al. 2016).

If an entomopathogenic fungus is used for pest control in the field, it must exert satisfactory insecticidal activity under a variety of environmental conditions (Kubicek and Druzhinina 2007). Temperature is a key constraint restricting the ability and speed of which entomopathogenic fungi to infect and colonize host insects (Fargues et al. 2003, Yeo et al. 2003, Kryukov et al. 2018). Our results confirmed that 18–28°C was the optimum temperature for growth and sporulation of *M. hiemalis* BO-1. At 13°C and 33°C, growth and sporulation of *M. hiemalis* BO-1 were reduced in comparison with 23°C. A pathogenicity bioassay confirmed that *M. hiemalis* BO-1 also possessed stronger pathogenicity against *B. odoriphaga* larvae at 18–28°C. This agrees with other studies showing little germination and growth of *Metarhizium* spp at low temperatures (Ekesi et al. 1999, Bai et al. 2016b). The pathogenicity bioassay results were consistent with the biorational assay results. At 13°C *M. hiemalis* BO-1 exerted a slightly more pathogenic effect than that at 33°C. It is possible that the low temperature merely slowed the pathogenic process of the fungus while the high temperature blocked the pathogenic process. Similarly, Fargues and Luz (2000) and Soyelu et al. (2020) reported that a temperature between 20 and 25°C was optimum for the pathogenicity of *B. bassiana* against *Rhodnius prolixus* nymphs and *Musca domestica* larvae and adults, and the fungal virulence declined rapidly at temperatures exceeding 30°C. This indicated that extreme temperatures not only restricted the growth and infection of the fungus, but also accelerated nutrient metabolism resulting in less extracellular enzymes and toxic proteins (Bai et al. 2016b). Previous studies reported that during the emergence peak of *B. odoriphaga* larvae, the mean soil temperature fluctuates between 15 and 25°C, which would be suitable for the use of *M. hiemalis* BO-1 to control *B. odoriphaga* based on our results (Shi et al. 2018, 2020). To sum up, *M. hiemalis* BO-1 application could provide satisfactory control efficiency during the spring and autumn in open fields, or during winter in the greenhouse. During these seasons, the soil temperature ranges between 10 and 25°C (Shi et al. 2018). After summer and winter, *M. hiemalis* BO-1 reapplication could increase the soil abundance of this fungus and increase its efficacy. However, the pathogenicity of entomopathogenic fungi is also dependent on humidity and other abiotic factors. In general, dry conditions and poor nutrition are generally unsuitable for the propagation of fungi (Garrido-Jurado et al. 2011). Further studies on the interactive relationships among temperature, humidity, and the pathogenicity of fungi should be conducted.

Many entomopathogenic fungi exhibit good insecticidal activity in the laboratory, but the field control was unsatisfactory. This performance failure has been attributed to complex natural environment conditions (Hussein et al. 2010, Garrido et al. 2011). We simulated field experiments using potted Chinese chives plants to evaluate the potential of *M. hiemalis* BO-1 for the control of root maggots. The results confirmed that when the concentration of spore suspension exceeded 1 × 10^7^ spores/ml, the control efficiency was satisfactory. At 5 d after treatment, the control efficiencies were 81.28% (1 × 10^7^ spores/ml) and 93.58% (1 × 10^9^ spores/ml) for 2nd instar larvae, and 70.16% (1 × 10^7^ spores/ml) and 84.29% (1 × 10^9^ spores/ml) for 4th instar larvae. This is an exceptional result compared to other entomopathogenic fungi, which display excellent control efficiency in the field only when the concentration of the spore suspension exceeds 1 × 10^11^ spores/ml (Zhou et al. 2014, Coombes et al. 2016, Murigu et al. 2016). *M. hiemalis* BO-1 possessed excellent control efficiency against *B. odoriphaga* larvae, and control was rapid, with only three 3–5 d being was required to achieve a high level of control. Other biocontrol agents, such as *Beauveria bassiana* (Zhou et al. 2014), *Bacillus thuringiensis* (Song et al. 2016) and entomopathogenic nematodes (Wu et al. 2017) require more than 7 d to achieve the same effect. However, combinations with other entomopathogenic fungi or chemical insecticides could provide better control and these should be studied further.

In conclusion, we isolated a new entomopathogenic fungi strain from infested *B. odoriphaga* larvae. This was identified as *Mucor hiemalis* BO-1 based on the morphological and molecular characteristics. Bioassay results confirmed that *Mucor hiemalis* BO-1 exhibited the greatest pathogenicity to *B. odoriphaga* larvae at 18–28°C, a temperature range that was beneficial to fungal growth and sporulation. A pot experiment confirmed that *M. hiemalis* BO-1 possessed efficient control efficiency against *B. odoriphaga* larvae, and control exceeded 80% within 5 d. *M. hiemalis* BO-1 should be further evaluated for use as a biocontrol agent for *B. odoriphaga*.
